# Insights on the Optical Properties of Estuarine DOM – Hydrological and Biological Influences

**DOI:** 10.1371/journal.pone.0154519

**Published:** 2016-05-19

**Authors:** Luísa Santos, António Pinto, Olga Filipe, Ângela Cunha, Eduarda B. H. Santos, Adelaide Almeida

**Affiliations:** 1 Department of Biology & CESAM, University of Aveiro, Campus de Santiago, 3810–193 Aveiro, Portugal; 2 Research Centre for Natural Resources, Environment and Society (CERNAS), College of Agriculture, Polytechnic Institute of Coimbra, Bencanta 3045–601 Coimbra, Portugal; 3 Department of Chemistry & CESAM, University of Aveiro, Campus de Santiago, 3810–193 Aveiro, Portugal; NSYSU, TAIWAN

## Abstract

Dissolved organic matter (DOM) in estuaries derives from a diverse array of both allochthonous and autochthonous sources. In the estuarine system Ria de Aveiro (Portugal), the seasonality and the sources of the fraction of DOM that absorbs light (CDOM) were inferred using its optical and fluorescence properties. CDOM parameters known to be affected by aromaticity and molecular weight were correlated with physical, chemical and meteorological parameters. Two sites, representative of the marine and brackish water zones of the estuary, and with different hydrological characteristics, were regularly surveyed along two years, in order to determine the major influences on CDOM properties. Terrestrial-derived compounds are the predominant source of CDOM in the estuary during almost all the year and the two estuarine zones presented distinct amounts, as well as absorbance and fluorescence characteristics. Freshwater inputs have major influence on the dynamics of CDOM in the estuary, in particular at the brackish water zone, where accounted for approximately 60% of CDOM variability. With a lower magnitude, the biological productivity also impacted the optical properties of CDOM, explaining about 15% of its variability. Therefore, climate changes related to seasonal and inter-annual variations of the precipitation amounts might impact the dynamics of CDOM significantly, influencing its photochemistry and the microbiological activities in estuarine systems.

## Introduction

In aquatic systems, dissolved organic matter (DOM) has a fundamental ecological role, serving as nutrient source for heterotrophic and autotrophic organisms, absorbing light at surface waters, and interacting as a reactant, sorbent and chelator with anthropogenic compounds and metal ions [1]. DOM comprises a complex, heterogeneous continuum from high- to low-molecular-weight compounds that exhibit different water solubilities and reactivities [2]. The molecular size and chemical structures of DOM influence its bioavailability and nutritive value [3–5].

The estuary is the transition zone that links the terrestrial to the oceanic environments. DOM in estuaries is comprised of organic materials derived from a diverse array of both allochthonous and autochthonous sources. Riverine inputs, autochthonous production from algal and vascular plants, benthic fluxes, groundwater inputs, and exchange with adjacent coastal systems are the major sources of DOM in estuaries [6]. The source of DOM may have influence on its bio- and photochemical reactivity [7–9].

The fraction of DOM that absorbs light in a broad range of UV and visible wavelengths is designated by chromophoric dissolved organic matter (CDOM) [10, 11]. The absorption and fluorescence spectroscopic properties of CDOM have been extensively used to characterize and to infer about the source in a broad range of aquatic systems [12–17]. UV-absorbance characteristics of CDOM can be used to infer on the amount of DOM [18], aromatic content [7, 19–21] and average molecular weight [22], as well as, bio and photochemical reactivity [14, 23, 24]. Besides absorbing light (being coloured), CDOM also fluoresces when excited by light in the UV and blue regions of the spectrum. The two major classes of DOM components that have been found to fluoresce are humic-like materials and protein fractions [13, 15]. The presence and the relative intensities of these components vary according to the type, origin and concentration of DOM and therefore their motorization is suitable to follow seasonal and spatial changes. Furthermore, fluorescence techniques are more sensitive than absorption spectroscopy and both excitation and emission spectra show greater detail and provide more information as to chemical composition than do absorbance spectra [10].

This study aimed to characterise the seasonal profiles of variation and to identify the main sources of CDOM in an estuarine system (Ria de Aveiro). For that, selected CDOM optical and fluorescence properties, partly influenced by aromaticity and molecular weight, were correlated with physical, chemical and meteorological parameters.

## Methods

No specific permits were required for the described field studies. Our study area is not privately owned. The study did not involve endangered or protected species.

### Study site and sampling

**Fig 1:**
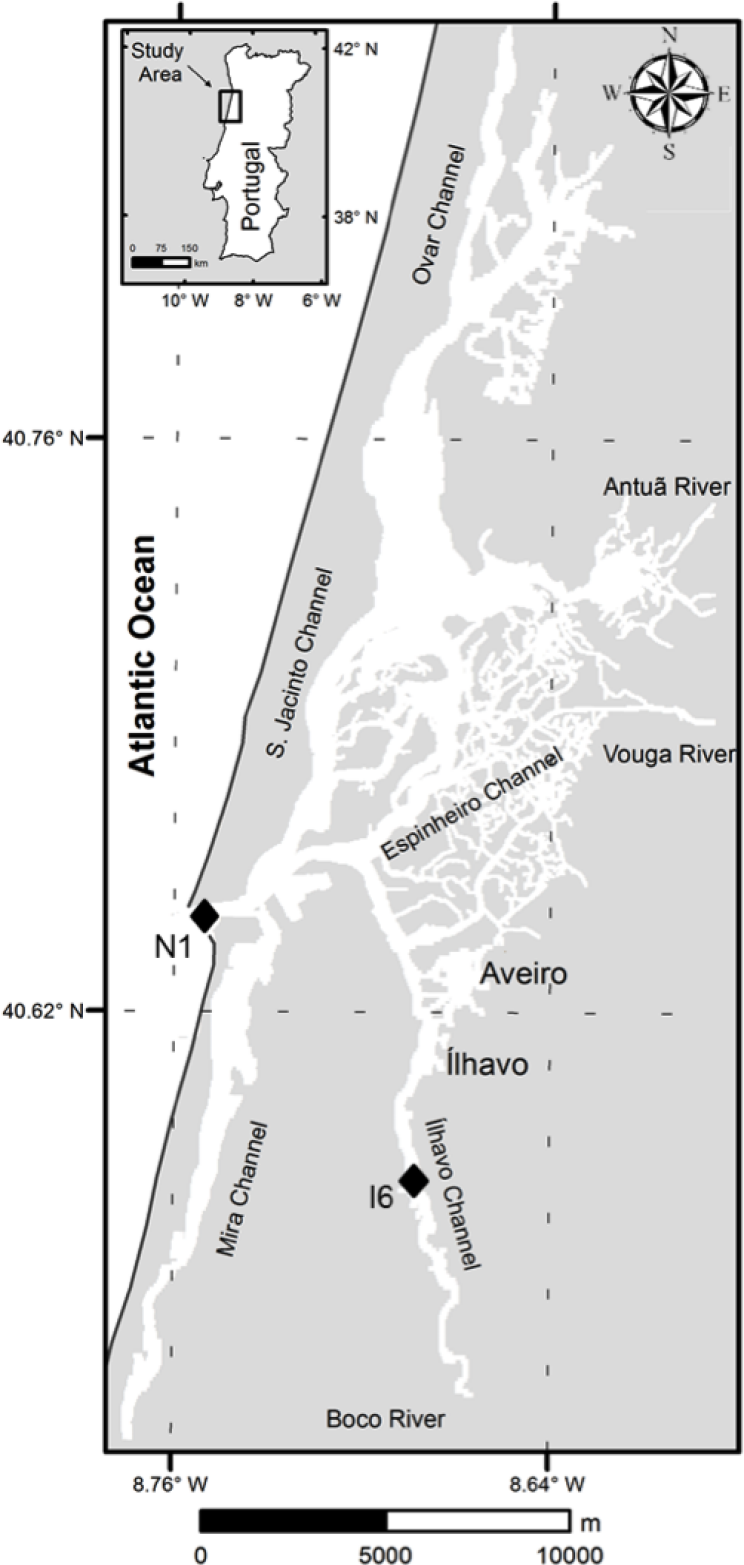
The estuarine system Ria de Aveiro with indication of sampling stations. Station N1 in Canal de Navegação represents the marine zone, and station I6, in Canal de Ílhavo, represents the brackish water zone. Reproduced by permission of The Royal Society of Chemistry (RSC) for the European Society for Photobiology, the European Photochemistry Association, and the RSC.

Ria de Aveiro (40° 38’N, 8° 45’W; Fig 1) is a shallow tidal lagoon [25] situated on the Northwest Atlantic coast of Portugal, separated from the sea by a sand bar. The lagoon covers an area ranging from 66 at low tide to 83 km^2^ at high tide. It exchanges with the sea a volume of water of 137 Mm^3^ for maximum spring tide and 35 Mm^3^ for minimum neap tide [25]. The lagoon has a complex topography, with four main channels spreading from the mouth: S. Jacinto, Espinheiro, Mira and Ílhavo. Due to their unique characteristics, each channel could be considered as an independent estuary connected to a common inlet [26]. Freshwater is supplied to lagoon mainly by rivers Vouga, Antuã, Caster, Gonde and Boco, which discharge an average water input of 1.8 Mm^3^ during a tidal cycle [27]. There are only two real-time fresh water stations from Sistema Nacional de Informação de Recursos Hídricos (SNIRH) located in Vouga and Antuã basins, recording periodically river flow data. The data available is measured far from the lagoon, and is scarce and incomplete; thus freshwater inputs to the lagoon have to be estimated from the climatological analysis [28]. Therefore, in the present study, we will use the cumulative precipitation data for the prior three weeks to estimate the freshwater inputs in the estuary. The 21-days cumulative precipitation showed the best correlation with freshwater inputs and adequate to predict the hydrological environment in this estuarine system [29].

Two estuarine sites (stations N1 and I6; Fig 1) were surveyed regularly (26 times), between 17^th^ May 2011 and 31^st^ January 2014, always at low tide. The two sites display distinct levels of microbial activity [30–33], concentration of particulate and dissolved organic matter [17, 29] and are differently impacted by river discharges and oceanic influence [29]. Station N1, located near the mouth of estuary, is highly exposed to oceanic influence, whereas station I6, located at the inner section of the Ílhavo channel, the narrowest and shortest of the main channels [26], is directly influenced by river Boco discharge. Previous simulations of the influence of freshwater inputs on the water residence time in the estuary showed that under a scenario of maximum freshwater inflow, water masses in the estuary have very low residence time. In opposition, during a minimum freshwater inflow situation, water masses showed a long residence time, remaining in the estuary for long time [29]. Surface water samples, approximately at 20 cm depth, were collected with a bucket, transferred to pre acid-washed dark glass 3 L flasks and transported in the dark in cool boxes at approximately 4°C to the laboratory and processed immediately. The characterization of samples included not only the UV-visible and fluorescence spectroscopic characterization but also the determination of nutrients concentrations and dissolved organic carbon. In some samples, it was not possible to make all the analysis; thus, the number of samples may be different for different parameters.

### Meteorological conditions and water column properties

Precipitation and solar irradiance data prior to sampling events were recorded at the meteorological station of the University of Aveiro, located on the vicinity of sampling sites. Water temperature and salinity were measured in the field using a WTW LF 196 Conductivity Meter (Wissenschaftlich Technische Werkstätten, Weilheim, Germany).

### Inorganic nutrients

For nutrient analysis, water subsamples previously filtered by 0.22 μm pore size (Durapore, Merck Millipore, USA) were stored at -20°C in acid-cleaned borosilicate flasks until determination. Nitrite and ammonia were quantified using methods described by Hansen and Koroleff [34]. Nitrate was assayed using an adaptation of the spongy cadmium reduction technique [35], with the nitrite value subtracted from the total.

### Dissolved organic carbon (DOC)

For DOC analysis, water subsamples were filtered through 0.22 μm pore size membranes (Durapore, Merck Millipore, USA), acidified to pH 2 with 2% (v/v) of HCl (2M) and stored at -20°C in acid-cleaned borosilicate flasks until determination. After thawed and immediately before analysis, subsamples were sparged with N_2_ during 5 minutes in order to remove inorganic carbon. The concentrations of total organic carbon (TOC) were determined by high temperature catalytic oxidation (HTCO) using a Shimadzu TOC-V_CSH_ at 680°C, flux gas flux (O_2_) of 150 mL min^-1^ with platinized-alumina catalysts (0.5% Pt on an alumina support) and a non-dispersive infrared detector (NDID) [36]. A standard curve for TOC concentration was made with potassium hydrogen phthalate in the range of 0 to 10 ppm. The correlation coefficients of calibration curves were equal or better than r = 0.999 and the precision of the analysis, expressed as a relative standard error, was below 10% for 0.5 ppm.

### Dissolved carbohydrates analyses

Monosaccharides (MCHO) and polysaccharides (PCHO) were determined by the 2,4,6-tripyridyl-s-triazine (TPTZ, Sigma) spectrophotometric method [37]. Succinctly, 1 ml of sample was mixed with 1 ml of potassium ferricyanide solution (0.7 mM) and kept in a boiling-water bath for 15 min. One millilitre of ferric chloride solution (2 mM) and 2 mL of TPTZ (2.5 mM) were added immediately after, tubes were vortexed and kept in the dark for 30 min. Then the absorbance was measured at 595 nm with a UV–visible spectrophotometer Shimadzu, UV 2101 PC model, using 1 cm quartz cell. The concentrations of total dissolved carbohydrates (TCHO) were measured by this method after acid hydrolysis performed in Teflon capped glass test tubes with 4 ml of sample and 0.4 ml HCl (1 M) at 150°C for 1 h. After hydrolysis, tubes were let to cool at room temperature and 0.4 ml of NaOH (1M) was added to neutralize the sample. TCHO was determined using a 1 ml of hydrolysate as described above for MCHO. The concentration of TCHO was corrected to the dilution factor by multiplying the final concentration by 1.2.

The concentrations of MCHO and TCHO were directly calculated based on a calibration curve (0.5 to 4 ppm) made from D-(**+**)-Glucose (Sigma) after subtracting the absorbance of the blank (Milli-Q water). Blank and standards were treated as samples for MCHO analysis. Glucose equivalents (μM) were converted into carbon (μM C) multiplying by a factor of 6 (according to the molecular structure of glucose) assuming that all monosaccharides in the samples were hexoses [37]. Correlation coefficients for our calibration curves were equal or better than r = 0.999. The concentration of PCHO is equal to the difference between the concentrations of TCHO and MCHO ([PCHO] **=** [TCHO]—[MCHO]). The solutions were protected from light during the whole analytical procedure.

### CDOM spectroscopic characteristics

#### UV-visible absorption spectroscopy

UV–Visible spectra of CDOM in water samples were obtained on a UV–visible spectrophotometer Shimadzu, UV 2101 PC model using 1, 5 or 10 cm quartz cuvettes (depending on the absorbance of samples) in the range 200–800 nm. The absorption coefficients (a_λ_, m^− 1^) at each wavelength (λ) were calculated as a_λ_ = 2.303 A_λ_/l, where A_λ_ is the absorbance reading at wavelength λ and l (m) is the optical path length [38]. Ultrapure water was used as reference and each spectrum was corrected for spectral offset by subtracting the average apparent absorbance from 700 to 800 nm [38].

The absorption coefficients at 250 and 350 nm were used for the determination of the spectral characteristics of CDOM. The absorption coefficients at 250 nm (a250) has been used as a proxy for allochthonous DOM [39] and at 350 nm (a350) has been used as a proxy for lignin phenols [20, 40]. The E_2_:E_3_ ratio is calculated as the ratio of a_250_ to a_365_ and was found to be inversely correlated with molecular size in humic acids isolated from Finnish lakes [22]. Specific ultra-violet absorbance at 254 nm (SUVA_254_) was calculated by dividing the UV absorbance at 254 nm measured in inverse meters (A_λ_/l) by the DOC concentration in mg L^-1^. SUVA_254_ is indicative of the amount of humification or aromaticity within the sample [19].

#### Fluorescence spectroscopy

Excitation-emission matrices (EEMs) were recorded using a spectrofluorometer FluoroMax-4 (Horiba Scientific, USA) with a 1 cm quartz cuvette (four polished windows), run in sample emission to lamp reference mode (S/R). The excitation wavelengths (λ_ex_) spanned from 240 to 500 nm and emission wavelengths (λ_em_) from 290 to 600 nm, both in 5 nm increments. Excitation and emission slit widths were set to 10 nm, and the integration time was 0.1 s. Fluorescence data were corrected following the procedure described by Murphy et al. [41]. To correct S/R readings for instrument specific variations in spectral responses concerning to both excitation and emission wavelengths post analysis, manufacturer supplied correction factors were applied to convert S/R to the corrected Sc/Rc [42].

$$\frac{S_{c}}{R_{c}}=\frac{C_{em}\times S}{C_{exc}\times R}$$

where C_em_ and C_exc_ are the correction factors for the emission and the excitation, respectively. EEMs of samples were corrected for primary and secondary inner filter effects, following a procedure proposed by Lakowicz [43], and whose efficacy has been assessed elsewhere [44]. The inner filter correction factor (IFCF) was calculated according to Eq (1)

$$IFCF=10^{0.5\times (A_{ex}+A_{em})}$$ (1)

where A_ex_ and A_em_ are the absorbances at the excitation and emission wavelengths for a pathlength of 1 cm. EEMs were blank-subtracted using the EEM of Milli-Q water.

Daily lamp and water-Raman checks were done. The lamp check is an excitation scan and serves the purpose of calibrating for excitation by verifying that the maximum intensity is at the correct wavelength for a xenon lamp (e.g., around 467 nm). The daily water-Raman scan serves to calibrate for emission wavelength verifying if the water Raman peak maximum occurred at 397 nm.

The sample EEMs and blanks were normalized to water Raman peak area dividing the intensities by the Raman area of the pure water emission spectrum recorded in the same day as samples, integrated over a λ_em_ range of 381 to 426 nm, at a λ_ex_ of 350 nm [45]. In order to convert the spectra to quinine sulfate units, a stock solution of quinine sulfate di-hydrate (Sigma) was made in H_2_SO_4_ (0.05M). Standards of quinine sulfate with concentrations in the range 0–45 ppb were prepared by dilution of the stock solution. The fluorescence intensities of those solutions were measured at λ_ex_350 / λ_em_450. A calibration line with r = 0.999 was obtained. The slope of that calibration line was normalized dividing by the Raman area of the water spectrum of that day. All the EEM spectra were converted from Raman units to quinine sulfate units (QSU) dividing by the normalized slope of the calibration line for quinine sulfate.

The signal to noise ratio for the Fluoromax-4 fluorometer was 7114:1 (±1742), for the water-Raman peak at an excitation wavelength of 350 nm. The signal to noise ratio was calculated as the difference in the peak intensity (at emission wavelength 397 nm) and background signal (at emission wavelength 450 nm), divided by the square root of the background signal (according to operation manual) [46].

The major peaks in the EEMs were identified using the wavelength ranges of the fluorescence peaks classically defined in natural DOM fluorescence spectra. The wavelength ranges and descriptors of those peaks are presented in Table 1. In the present paper the nomenclature of Parlanti et al. [47] was adopted, but its correspondence to the nomenclature of Coble [15] is also presented in Table 1. The fluorescence peaks may present small variations in their positions within the spectral domains presented by Parlanti et al [47], but in the present work, in order to compare more easily the results obtained with different samples, the fluorescence intensities of the five peaks were determined at fixed excitation/emission wavelengths shown in Table 1.

**Table 1:** Major fluorescence bands in water, with notations used herein (Parlanti et al. [47]) and nomenclature proposed by Coble et al. [15].

Band (Parlanti et al. [47])	Excitation max. (nm)	Emission max. (nm)	Fluorophore type	Letter used by Coble et al. [15]	λ_Exc_/λ_Em_ (used in the present work)
**α**	330–370	420–480	Humic-like	C	320/440
**α′**	230–260	380–480	Humic-like + recent materials	A	260/425
**β**	310–320	380–420	Marine Humic-like; Autochthonous production	M	315/400
**γ**	270–280	300–340	Protein-like (Tyrosine-like)	B	280/310
**δ**	225–237	340–381	Protein-like (Tryptophan-like)	T	280/340

Two fluorescence indices were determined: the humification index (HIX), which is associated to the degree of humification (HIX) of DOM, and the index of recent autochthonous contribution (BIX), which allowed to assess the autotrophic productivity. These indices are useful tools for readily defining and classifying DOM characteristics in estuarine waters [16].

The HIX index was adapted from studies regarding DOM in soil [48] and introduced by Huguet et al. [16] in aquatic ecosystems for the study of the complexity of DOM dynamics in estuaries. The HIX was calculated as the ratio H/L of two spectral region areas from the emission spectrum scanned for excitation at 255 nm. These two areas are calculated between emission wavelengths 300 nm and 345 nm for L and between 435 nm and 480 nm for H. When the degree of aromaticity of DOM increases, the emission spectrum (at λ_ex_ 255 nm) is red shifted, which implies that the H/L ratio, and thus the HIX index, increases [16, 48]. The BIX index was introduced by Huguet et al. [16] to determine the presence of the β fluorophore, characteristic of autochthonous biological activity in water samples. BIX is calculated at λ_ex_ 310 nm, by dividing the fluorescence intensity emitted at λ_em_ 380 nm, corresponding to the maximum of intensity of the β band when it is isolated, by the fluorescence intensity emitted at λ_em_ 430 nm, which corresponds to the maximum in the α band.

### Data analysis

**Fig 2:**
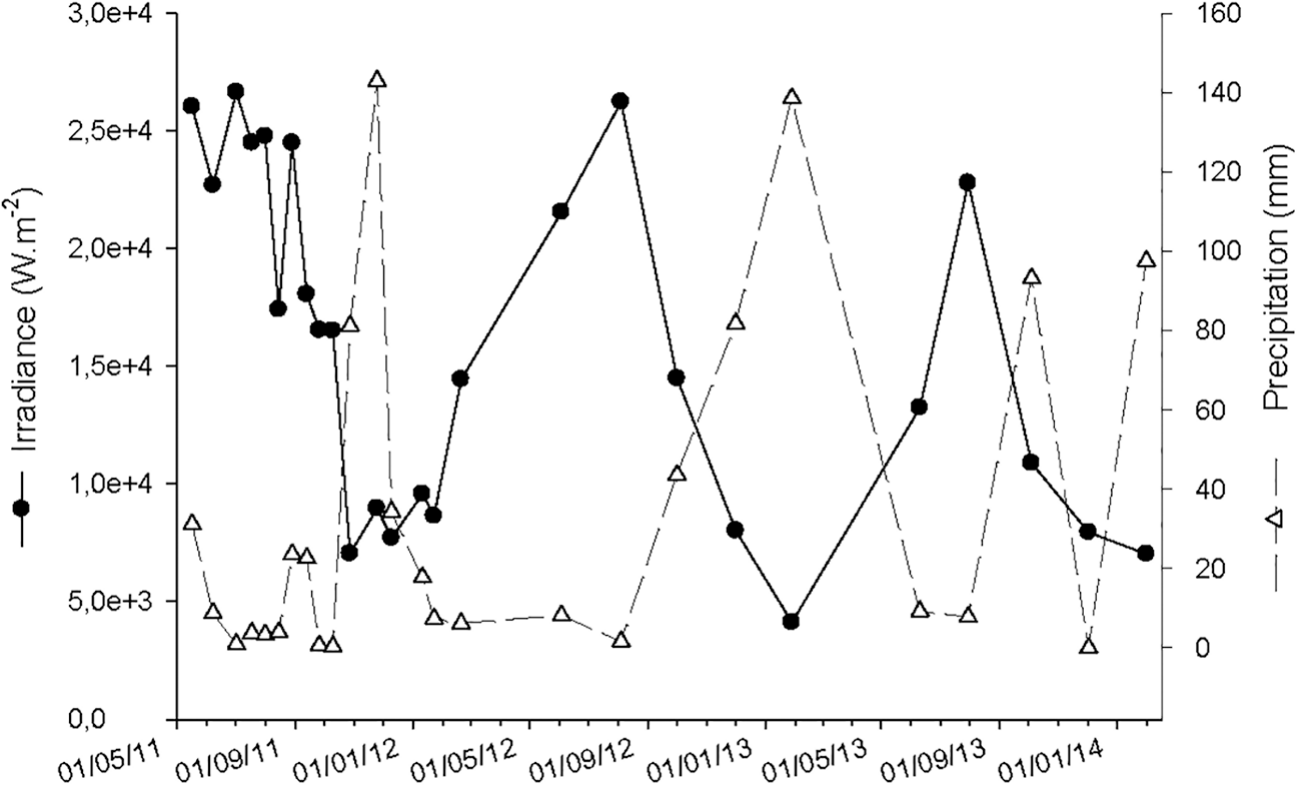
Daily average solar irradiance (3 days) and 21-days cumulative precipitation values in Ria de Aveiro, prior to the sampling events.

**Fig 3:**
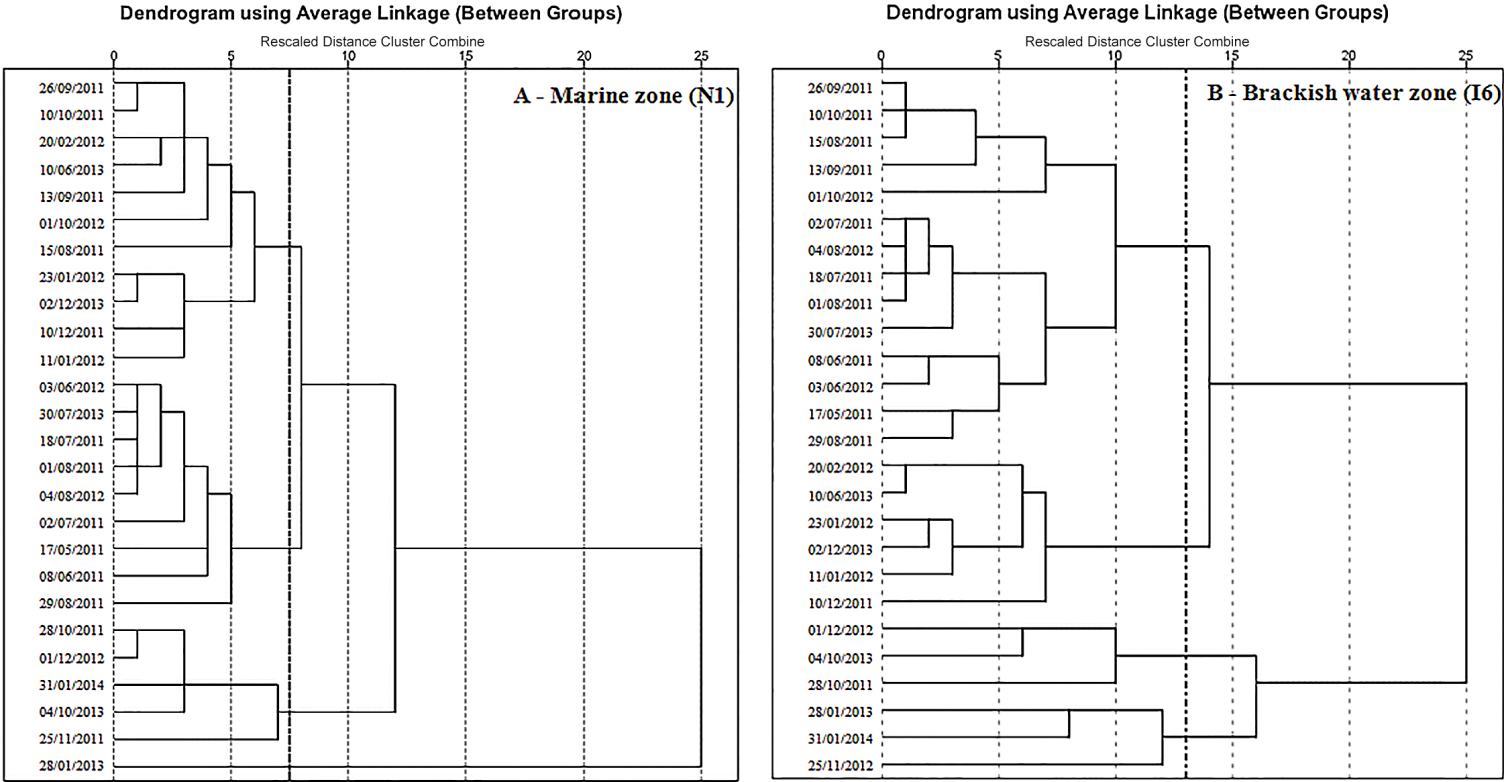
**Dendrograms constructed on Euclidean distances between sampling events obtained by hierarchical cluster analysis of salinity, precipitation and solar irradiance data at the marine (A) and brackish water zone (B) of the estuarine system**.

The statistical analysis of data was performed with the SPSS 17.0 (SPSS Statistics) software. Normal distribution was assessed by the Kolmogorov-Smirnov test and homogeneity of variation by the Levene test. Salinity, precipitation and solar irradiance data obtained during the diverse sampling events (Table 2 and Fig 2) were clustered using hierarchical cluster analysis (HCA). The euclidean distance was used to determine the similarity between-groups and values were standardized to z scores. The dendrograms are presented in Fig 3, where minimum rescaled distances of 7.5 in the case of marine zone (station N1) and 13 in the case of brackish water zone (station I6) were used as criteria to establish clusters individuality. The significance of differences in optical properties of DOM between the two estuarine zones was determined by the Mann-Whitney test and between the different groups by the Kruskall-Wallis test, followed by post-hoc multiple comparisons [49]. The relations between the different parameters were examined using a Spearman correlation. All physical and chemical parameters, as well as DOM properties were used for perform principal component analysis (PCA). The Kaiser-Meyer-Olkin (KMO) was used to test sampling adequacy and the Bartlett's Test to assess sphericity. After the analysis of the scree-plot, PCA was set to extract 2 components by the orthogonal rotation method Varimax [50]. For station N1, N was 83 and KMO = 0.636 and, for station I6, N was 92 and KMO = 0.798. For both stations the Bartlett's Test < 0.001.

**Table 2:** Physical and chemical properties at the surface of the water column in the marine (station N1) and brackish water (station I6) zones of the estuarine systems Ria de Aveiro during the sampling events.

Year	2011													2012							2013					2014
Month	May	Jun	July		Aug			Sep		Oct		Nov	Dec	Jan		Feb	Jun	Aug	Oct	Dec	Jan	Jun	Jul	Oct	Dec	Jan
Day	17	8	2	18	1	15	29	13	26	10	28	25	10	11	23	20	3	4	1	1	28	10	30	4	2	31
Marine zone (N1)																										
Temp (°C)	18.0	18.0	18.0	22.0	16.0	18.0	18.0	20.0	17.0	16.0	16.0	15.0	15.0	12.0	13.0	10.0	17.0	18.0	19.0	13.0	13.0	17.0	21.0	20.0	11.0	13.0
Sal	34.0	33.0	36.0	35.0	35.0	37.0	27.0	35.0	35.0	35.0	34.0	32.0	34.0	34.0	35.0	34.0	35.0	35.0	35.0	34.0	22.0	35.0	35.0	33.0	35.0	33.0
pH	8.1	8.2	8.1	8.1	8.1	8.0	7.9	7.9	7.9	7.8	7.9	8.3	8.4	8.5	8.3	8.4	8.3	8.2	8.2	7.9	8.3	8.5	8.3	7.9	7.9	8.0
Brackish water zone (I6)																										
Temp (°C)	21.0	22.0	22.0	25.0	21.0	23.0	22.0	22.0	21.0	18.0	15.0	13.0	12.0	10.0	11.0	8.0	23.0	23.0	20.5	12.0	11.5	19.0	26.0	21.0	8.0	12.0
Sal	27.0	25.0	35.0	34.0	36.0	35.0	30.0	35.0	35.0	34.0	30.0	14.0	21.0	24.0	25.0	27.0	27.0	36.0	34.0	15.0	0.0	28.0	35.0	21.0	27.0	4.0
pH	7.7	7.9	7.8	7.9	7.8	7.8	7.7	7.8	7.6	7.8	7.3	8.0	8.3	8.4	7.7	8.3	8.2	8.1	7.9	7.6	7.7	8.2	7.7	7.4	7.8	7.9

Temp–temperature; Sal—salinity

## Results

### Meteorological parameters

The irradiance and precipitation values (Fig 2) along the two-year survey showed a seasonal pattern of variation, typical of a Southeastern Europe country, where the highest values of irradiance are registered in spring/summer and of precipitation in autumn/winter seasons. Prior to sampling events, the daily average irradiance (3 days) varied between 4.13 and 26.6 x 10^3^ W m^-2^ (average 15.4 ± 6.93 x 10^3^ W m^-2^). Ranging from 0 to 143 mm, the 21-days cumulative precipitation values showed 4 main peaks, corresponding to the sampling events on 25/11/2011, 28/01/2013, 4/10/2013 and 31/01/2014.

### Water column properties

The temperature, salinity and pH determined during the different sampling events at the marine (station N1) and brackish water (station I6) zones of the estuary Ria de Aveiro are presented in Table 2. Water temperature varied between 10 and 22°C (average ± standard deviation, 16.3 ± 3.1°C) at the marine zone (station N1) and between 8 and 26°C (average 17.7 ± 5.6°C) at brackish water zone (station I6). Salinity ranged from 22 to 37 (average 33.7 ± 3.0) at the marine zone (station N1) and from 0 to 36 (average 26.7 ± 9.6) at the brackish water zone (station I6). The variation of pH at both sampling sites was slight, averaging 8.12 ± 0.21 and 7.84 ± 0.25 at stations N1 and I6, respectively.

### Grouping of sampling events

Based on HCA, sampling events were clustered and the dendograms showed a clear structure comprising 4 individual groups (Fig 3) in both estuarine zones, which had dissimilar meteorological conditions and water column properties (Table 3). In both estuarine zones, group 1 contains the sampling events with highest salinity values, 34.95 ± 0.85 at the marine zone and 33.0 ± 3.4 at the brackish water zone, resulting from previous low precipitation values, less than 44 mm of cumulative precipitation in the previous 21 days, in both estuarine zones. In opposition, resulting from previous wet periods, with more than 98 mm of cumulative precipitation in the preceding 21 days, the group 4, in both estuarine zones, sets the sampling events when were registered the lowest values of salinity. In the marine zone, the group 4 contained a unique sampling event, which happened immediately after a period of heavy rain and storm conditions, conferring particular characteristics to the water column.

**Table 3:** Physical and chemical properties at the surface of the water column and, meteorological environment, within the different groups in the marine (station N1) and brackish water (station I6) zones of the estuarine systems Ria de Aveiro.

Groups	1	2	3	4
Marine zone (N1)				
Salinity	35.0 ± 0.85	33.8 ± 2.6	33.2 ± 0.80	22 ± 0
	(34–37)	(27–36)	(32–34)	(22–22)
	N = 55	N = 34	N = 22	N = 4
Irradiance x 10^3^ (w.m^-2^)	13.4 ± 3.9	24.4 ± 1.7	8.4 ± 1.4	4.1 ± 0.0
	(7.7–18.1)	(21.5–26.6)	(7.02–10.9)	(4.1–4.1)
	N = 55	N = 34	N = 22	N = 4
Precipitation (mm)	12.8 ± 13.6	10.2 ± 10.2	100 ± 24	139 ± 0
	(0–44)	(1–31)	(81–143)	(139–139)
	N = 55	N = 34	N = 22	N = 4
Temperature (°C)	15.4 ± 3.2	18.5 ± 1.8	15.4 ± 2.5	13 ± 0
	(10–20)	(16–22)	(13–20)	(13–13)
	N = 55	N = 34	N = 22	N = 4
pH	8.14 ± 0.26	8.13 ± 0.12	7.98 ± 0.16	8.30 ± 0.0
	(7.8–8.47)	(7.89–8.3)	(7.88–8.26)	(8.3–8.3)
	N = 55	N = 34	N = 22	N = 4
Brackish water zone (I6)				
Sal	33.0 ± 3.4	25.2 ± 2.4	22.6 ± 6.6	6.62 ± 6.29
	(25–36)	(21–28)	(15–30)	(0–14)
	N = 61	N = 28	N = 13	N = 13
Irradiance x 10^3^ (w.m^-2^)	21.0 ± 4.1	10.2 ± 2.7	8.5 ± 1.7	6.9 ± 2.1
	(14.5–26.6)	(7.7–14.4)	(7.0–10.9)	(4.1–9.0)
	N = 61	N = 28	N = 13	N = 13
Precipitation (mm)	11.3 ± 12.9	13.1 ± 11.4	85.1 ± 5.6	128 ± 21
	(0–44)	(0–34)	(81–93)	(98–143)
	N = 61	N = 28	N = 13	N = 13
Temperature (°C)	22.0 ± 2.0	11.2 ± 3.6	15.9 ± 3.8	12.38 ± 0.51
	(18–26)	(8–19)	(12–21)	(12–13)
	N = 61	N = 28	N = 13	N = 13
pH	7.82 ± 0.14	8.10 ± 0.25	7.44 ± 0.13	7.84 ± 0.11
	(7.58–8.15)	(7.73–8.37)	(7.32–7.61)	(7.71–7.97)
	N = 61	N = 28	N = 13	N = 13

### Inorganic nutrients

**Fig 4:**
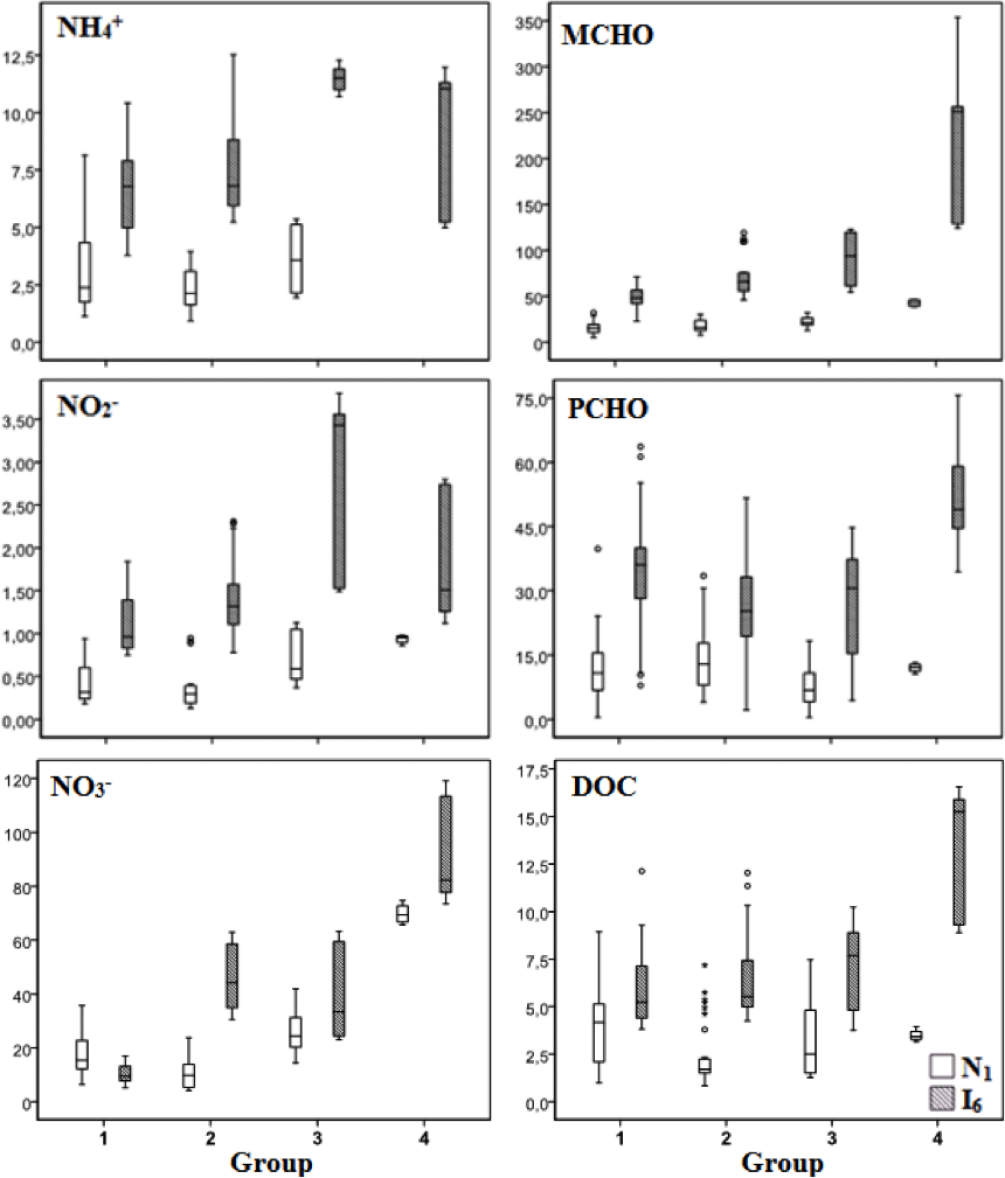
Boxplots of the concentrations of nitrate (μM), nitrite (μM), ammonium (μM), mono carbohydrates (MCHO, μM.C), polycarbohydrates (PCHO, μM.C) and DOC (mg L-1) within the different groups of samples at the marine (station N1) and brackish water (station I6) zones of the estuary Ria de Aveiro.

The concentration of inorganic nutrients within the different groups at the marine (station N1) and brackish water (station I6) zones of the estuary Ria de Aveiro is presented in Fig 4 and S1 Table. Values lower than the limit of detection (LOD) were not considered for the calculation of the averages presented in S1 Table. The number of samples within each group and the number with values lower than LOD is indicated in S1 Table.

As can be seen in the boxplots of Fig 4, the nutrients concentration tends to be higher in the brackish zone (I6) than in the marine zone (N1). The comparison between sampling sites, considering all the samples of each site, revealed a significant difference between the two sampling sites for nitrite and ammonia (Mann-Whitney test, p<0.05). The difference was not significant for nitrate, since the variation between groups of samples, at each sampling site, is large, and, thus, the range of values at one station overlaps the range at the other sampling station. Indeed, when only samples of one group are considered, the difference of nitrate concentration between sites is significant for each group of samples.

In what concerns the variation between groups, Fig 4 shows that the concentrations of nitrate and nitrite tend to increase from group 1 to group 4. That tendency was not observed for ammonia, perhaps due to the higher variability of the experimental data, within each group. The differences between the groups of samples are more clearly seen for nitrate. At the I6 station the difference between groups 2 and 3 is not significant but these groups are different from group 1 and group 4, with groups 2 and 3 exhibiting concentration values between these two extremes. At station N1 each group is different from the others, showing an increasing tendency from group 2 to group 4 and a small inversion of that tendency from group 1 to group 2.

### Dissolved organic carbon and carbohydrate species

The concentration of dissolved organic carbon (DOC), monosaccharides (MCHO) and polysaccharides (PCHO) within the different groups at the marine (station N1) and brackish water (station I6) zones of the estuary Ria de Aveiro are presented in S1 Table and Fig 4. The concentrations of carbohydrates (MCHO and PCHO) and DOC were significantly different between sampling sites, with higher values at the brackish zone (I6).

As can be seen in Fig 4, the monosaccharides concentrations tend to increase from group 1 to group 4, at station I6 (1<2≈3<4). That tendency is not so clear at station N1, but group 4 is significantly different from the other groups, presenting higher values. The polysaccharides concentrations were not significantly different between groups at station N1, but at station I6 there are significant differences between groups, with PCH concentrations from group 4 higher than those of the other groups.

In what concerns DOC concentrations, it was not possible to observe a tendency at station N1, probably due to the low values obtained and the high uncertainties associated. However, at station I6, DOC concentrations are higher for samples from group 4 (significant difference between group 4 and all the other groups)

### Chromophoric spectroscopic properties of DOM

**Fig 5:**
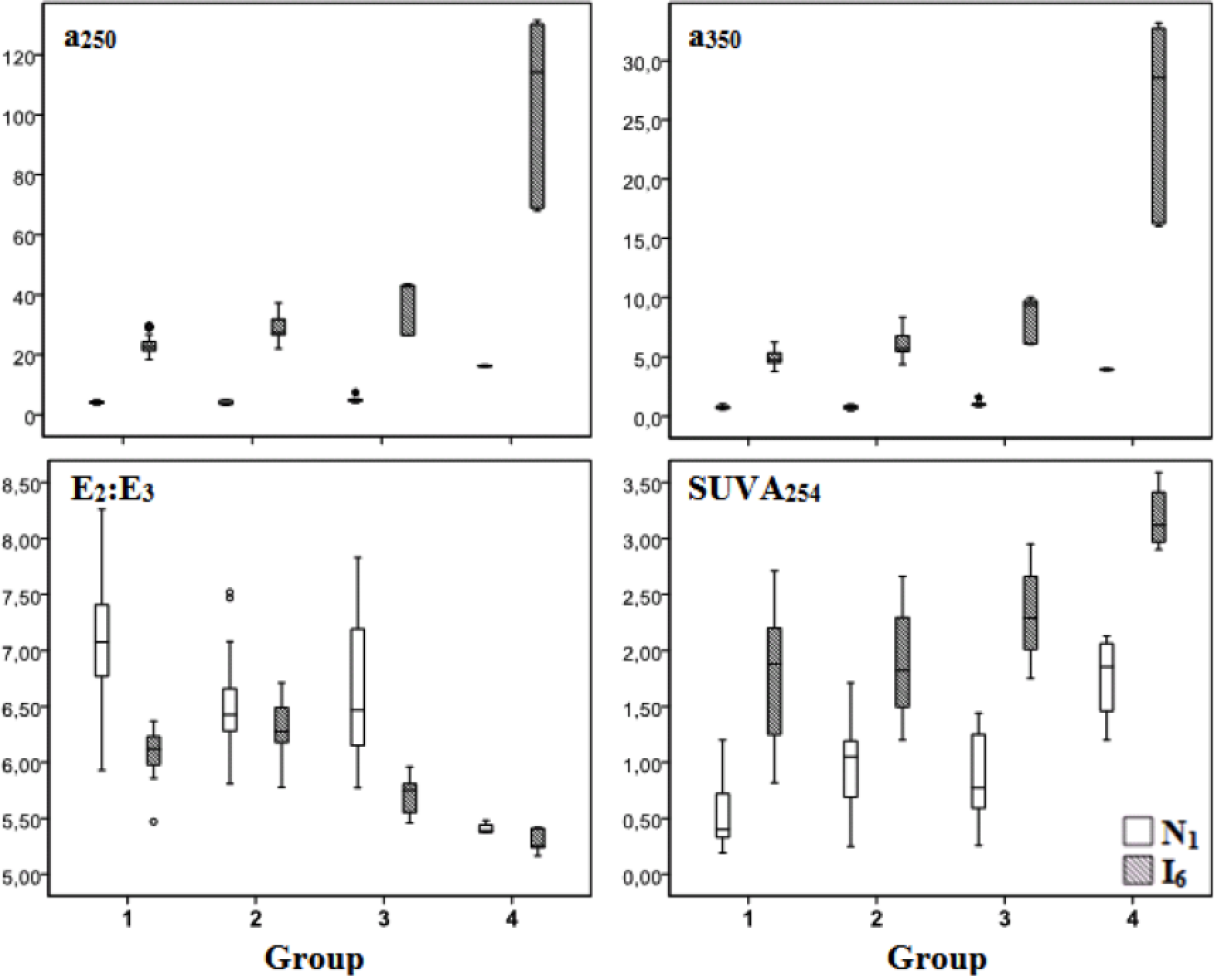
Boxplots of the UV-visible spectroscopic parameters a_250_ (m^-1^), a_350_ (m^-1^), E2:E3 ratio and SUVA_250_ (L mg^-1^ C^-1^ m^-1^) within the different groups of samples at the marine (station N1) and brackish water (station I6) zones of the estuary Ria de Aveiro.

The variations within the groups of the different spectroscopic properties of DOM at the marine (N1) and brackish water (I6) zones of the estuarine system Ria de Aveiro are presented in Fig 5 and S2 Table.

The absorption coefficients (a_350_ and a_250_) and SUVA_254_ differ significantly between sampling sites (Mann-Whitney test, p<0.05), presenting higher values at the brackish sampling site (I6) than at the marine zone (N1): a_350_, a_250_ and SUVA_254_ were, on average, 8.6, 7.4 and 2.6 times higher, respectively, at station I6 than at station N1. The ratio E_2_:E_3_ varied between 5.17 and 8.26 and was similar (Mann-Whitney test, p>0.05) at the stations N1 (average 6.8 ± 0.59) and I6 (average 6.0 ± 0.35), if samples of all groups are considered, since the range of values at one station overlaps the range at the other sampling station. However, when the data are split by groups, the E2:E3 values for groups 1, 2 and 3 are significantly different, being higher at station N1 than at station I6 (Mann-Whitney applied to data split by groups, p<0.05).

At the two stations, and for both absorption coefficients, the observed differences between groups were statistically significant (Kruskall-Wallis test, p<0.05). As can be seen in Fig 5, the absorption coefficients tend to increase from group 1 to group 4 both at station N1 (1≈2<3<4) and station I6 (1<2<3<4), with values for group 4, on average, at least 3 times higher than for the other groups, at both sampling sites. A similar increasing tendency from group 1 to group 4 is observed for SUVA_254_ at both stations.

The ratio E_2_:E_3_ was statistically different between the different groups of samples (Kruskall-Wallis test, p<0.05), describing a profile of variation which is approximately inverse of the variation of a_350_, a_250_ and SUVA_254_. The ratio E_2_:E_3_ tends to decrease from group 1 to group 4, with only an inversion of that tendency between groups 1 and 2 at station I6. At station N1, the difference of that ratio is not significant between groups 2 and 3 but these two groups are significantly different from the other two, exhibiting intermediate values between groups 1 and 4. Samples from group 4 exhibit the lowest values of E2:E3 at both sampling sites.

### Molecular fluorescence properties of DOM

**Fig 6:**
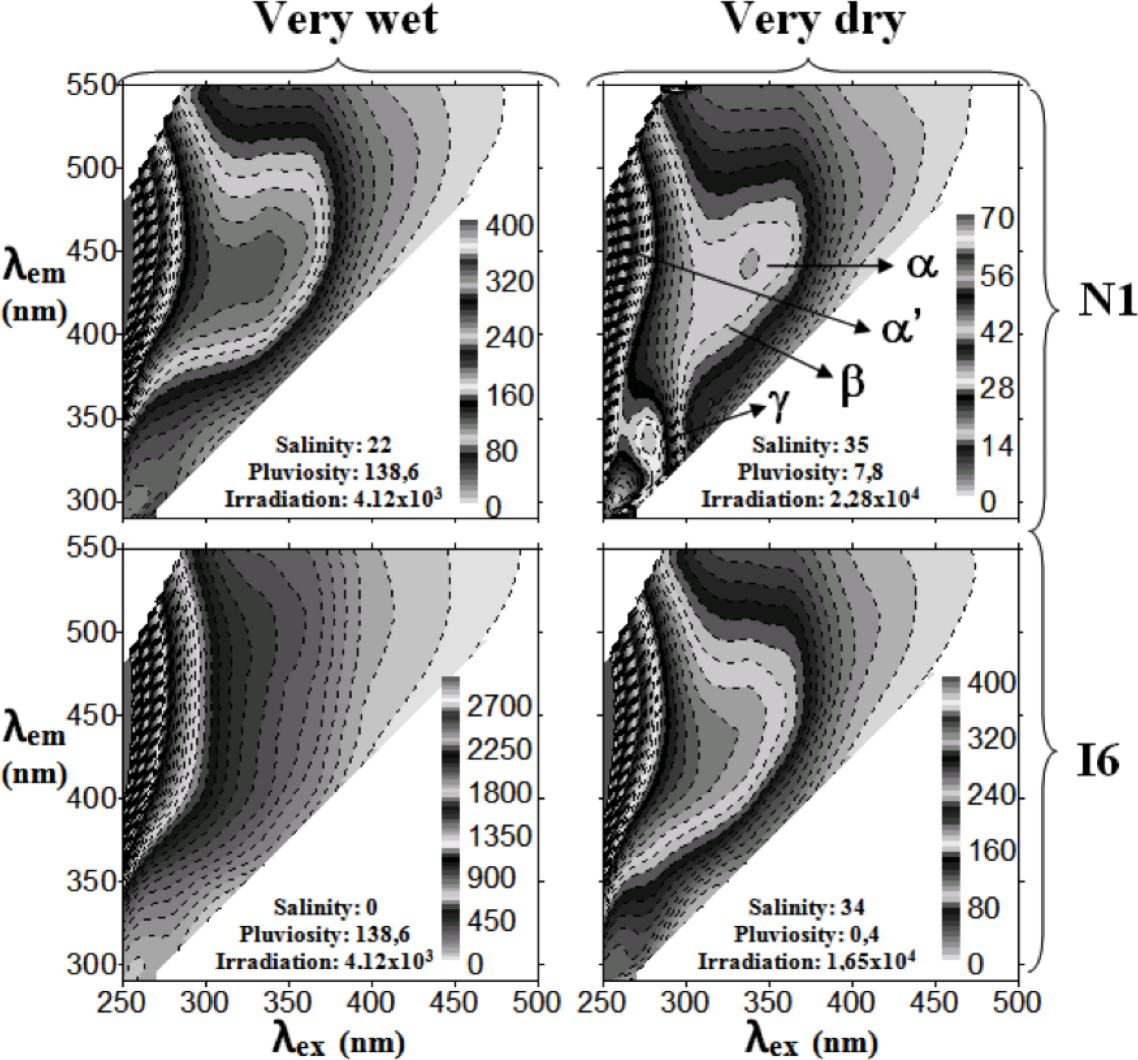
Contour EEM plots of 4 samples corresponding to group 1 (dry weather) and group 4 (very wet weather) from sampling stations N1 (marine zone) and I6 (brackish zone).

Fig 6 shows contour excitation-emission matrix (EEM) plots of EEM fluorescence spectra of 4 samples belonging to groups 1 and 4 of sampling events at sampling stations N1 (marine zone) and I6 (brackish zone). The location of the main fluorophores considered in the present work is indicated in one of the plots.

**Fig 7:**
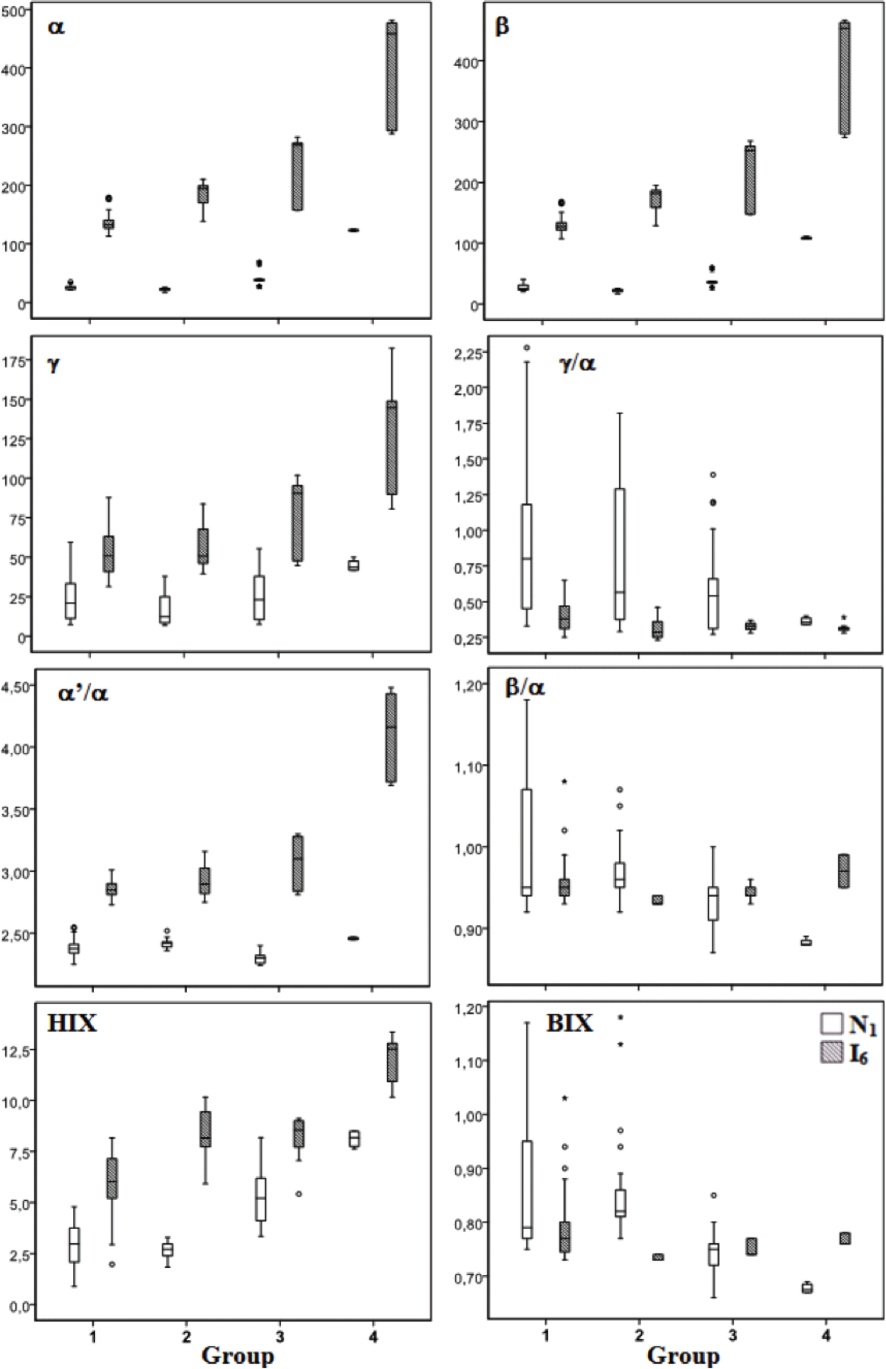
Boxplots of the intensities (in QSU) of several fluorophores (α, β and γ), of ratios between those intensities (γ/α, α’/α and β/α) and of fluorescence indexes BIX and HIX within the different groups of samples at the marine (station N1) and brackish water (station I6) zones of the estuary Ria de Aveiro.

The fluorescence intensity of the main fluorophores characteristic of DOM (α, α‘, β, γ and δ) in the water samples collected in the marine (station N1) and brackish water (station I6) zones of the estuarine system Ria de Aveiro within the different groups are shown in Fig 7 and S3 Table. Overall, the intensity of the main fluorophores of estuarine DOM was higher, varying between 2.9 and 8.2 times, at the brackish water station (I6) compared with marine station (N1).

Showing a similar pattern of variation, the fluorescence intensities of all these fluorophores were significantly different between sample groups (Kruskall-Wallis test, p<0.05) at both estuarine stations. An increasing trend of the intensities of these fluorophores is observed from group 1 to group 4. The highest intensities of all the fluorophores referred were observed in the group 4. The intensities of fluorophores α, α' and β in samples from αgroup 4 are up to 2.9 times higher at the marine zone (N1) and 1.8 times at brackish water zone (I6) compared with the other groups.

The absolute intensities of these fluorophores are influenced mainly by the concentration of fluorescent organic matter. Differences of composition are more clearly revealed by fluorescence indices and ratios, as described in the following section

### Fluorescence ratios and indices

The variation of the fluorescence ratios and indices within the groups at the marine (N1) and brackish water (I6) zones of the estuarine system Ria de Aveiro are shown in Fig 7 and S4 Table.

Considering all the samples of the four groups, the comparison between the two sampling sites revealed that they exhibit significant differences in the fluorescence ratios and indices of the dissolved organic matter (Mann-Whitney test, p<0.05). The ratio α'/α and the HIX index were higher at I6 sampling site (brackish zone), while the ratios γ/α and β/α and the BIX index were higher at the N1 sampling station (marine zone).

In what concerns variations between groups of samples, the ratio α‘/α showed a tendency of increase from group 1 to 4 at the brackish water zone (I6 station), as can be seen in Fig 7, but no clear tendency was observed at the marine zone (N1 station). Presenting a wider range of variation at the marine station (N1), particularly in group 1, the ratio γ/α exhibited significant differences between groups of samples (Kruskall-Wallis test, p<0.05). At both stations, the group 1 showed the highest values of the ratio γ/α, with significant differences between groups 1 and 4. At the marine zone (N1), the ratio γ/α decreased 2.5 times from group 1 to group 4. The ratio β/α showed higher variability (range 0.87–1.51) at the marine zone (N1) compared with brackish water zone (I6) (range 0.93–1.08), but this ratio presents significant differences between groups at both sampling stations (Kruskall-Wallis test, p<0.05), showing a decreasing tendency from group 1 to 4 at station N1 and higher values in the extreme groups (1 and 4) at station I6.

The variation of the BIX index between groups of samples was similar to the variation observed for the ratio β/α. Indeed, these two parameters are very highly correlated, as shown in the next section. Decreasing from the group 1 to 4, the BIX index varied significantly between groups at the marine station N1 (Kruskall-Wallis test, p<0.05). At the brackish water zone (I6), the BIX showed lower differences between groups and presented higher values in the extreme groups (1 and 4).

The HIX index showed significant differences between groups of samples and an increasing tendency from group 1 to group 4. At the marine zone (N1), the HIX varied greatly between groups (Kruskall-Wallis test, p<0.05), increasing 3 times from the group 2 to 4. At the brackish water zone (I6), the HIX index was also different between groups (Kruskall-Wallis test, p<0.05), increasing from group 1 to 2 and from 3 to 4.

### Spearman correlations

Generally, the spectroscopic properties of CDOM (Table 4) and the fluorescence peaks of FDOM (Table 5) correlated more strongly and with a higher number of parameters at the brackish water zone (I6) compared with marine zone (N1). The absorption coefficients of CDOM at 350 nm (a_350_) and at 250 nm (a_250_) were highly correlated at both estuarine sites (N1—p = 0.966; p = 0.00; N = 114 and I6- p = 0.931; p = 0.00; N = 115). At the I6 station, up of 75 and 60% of the variability of the absorption coefficients a_350_ and a_250_ were explained by salinity and precipitation, respectively. At the marine station (N1), the ratio E_2_:E_3_, in general, was not correlated with the studied variables, except with MCHO (negative correlation) and DOC (positive correlation). At the brackish water zone (I6), this ratio correlated positively with solar irradiance and salinity and negatively with precipitation, monosaccharides, nitrates and nitrites and DOC. SUVA_254_ correlated negatively with salinity and positively with precipitation at both estuarine stations. Besides, at station N1, SUVA_254_ correlated negatively with DOC, while in the brackish water zone it correlated positively with MCHO and nitrates and negatively with irradiance. At both stations, the intensities of α, α‘ and β peaks were correlated with all the parameters investigated. The γ peak correlated with the concentration of DOC at both estuarine stations. The intensities of γ and δ peak at station I6 were correlated with salinity, precipitation, monosaccharides and nitrate. The α‘/α at the marine zone (N1) (Table 6) was correlated with temperature, solar irradiance and nitrate concentration. At brackish water zone, this ratio correlated with salinity, solar irradiance, precipitation, monosaccharides, nitrate and DOC. The γ/α at marine zone was slightly correlated only with MCHO, whereas at the brackish water zone (I6) correlated with temperature, salinity, solar irradiance, monosaccharides and nitrate. The β/α correlated with a higher number of variables at marine zone (N1) compared with brackish water zone (I6), where was only correlated with temperature.

**Table 4:** Spearman correlation (p) between spectroscopic coefficients of CDOM, physicochemical parameters and, organic and inorganic nutrients.

CDOM		Temp.	Sal.	Irrad.	Prec.	MCHO	NO_2_^-^	NO_3_^-^	DOC
a_350_	N1	p = 0.053	p = -0.419^**^	p = -0.251^**^	p = 0.557^**^	p = 0.492^**^	p = 0.612^**^	p = 0.313^**^	p = 0.176
		(N = 114)	(N = 114)	(N = 114)	(N = 114)	(N = 87)	(N = 100)	(N = 113)	(N = 110)
	I6	p = -0.406^**^	p = -0.809^**^	p = -0.581^**^	p = 0.74^**^	p = 0.76^**^	p = 0.388^**^	p = 0.663^**^	p = 0.446^**^
		(N = 115)	(N = 115)	(N = 115)	(N = 115)	(N = 109)	(N = 110)	(N = 114)	(N = 112)
a_250_	N1	p = 0.082	p = -0.43^**^	p = -0.218^*^	p = 0.566^**^	p = 0.461^**^	p = 0.632^**^	p = 0.238^*^	p = 0.194^*^
		(N = 114)	(N = 114)	(N = 114)	(N = 114)	(N = 87)	(N = 100)	(N = 113)	(N = 110)
	I6	p = -0.464^**^	p = -0.851^**^	p = -0.569^**^	p = 0.699^**^	p = 0.771^**^	p = 0.347^**^	p = 0.693^**^	p = 0.478^**^
		(N = 115)	(N = 115)	(N = 115)	(N = 115)	(N = 109)	(N = 110)	(N = 114)	(N = 112)
E_2_:E_3_	N1	p = -0.163	p = 0.176	p = -0.127	p = -0.134	p = -0.299^**^	p = -0.073	p = 0.058	p = 0.237^*^
		(N = 114)	(N = 114)	(N = 114)	(N = 114)	(N = 87)	(N = 100)	(N = 113)	(N = 110)
	I6	p = -0.039	p = 0.299^**^	p = 0.357^**^	p = -0.647^**^	p = -0.471^**^	p = -0.404^**^	p = -0.274^*^	p = -0.206^*^
		(N = 115)	(N = 115)	(N = 115)	(N = 115)	(N = 109)	(N = 110)	(N = 114)	(N = 112)
SUVA_254_	N1	p = -0.006	p = -0.437^**^	p = 0.047	p = 0.376^**^	p = 0.063	p = 0.028	p = 0.087	p = -0.862^**^
		(N = 110)	(N = 110)	(N = 110)	(N = 110)	(N = 83)	(N = 96)	(N = 109)	(N = 108)
	I6	p = -0.173	p = -0.468^**^	p = -0.333^**^	p = 0.467^**^	p = 0.479^**^	p = 0.164	p = 0.409^**^	p = -0.226^*^
		(N = 112)	(N = 112)	(N = 112)	(N = 112)	(N = 109)	(N = 110)	(N = 114)	(N = 112)

Temp–Temperature; Sal–Salinity; Irrad–Irradiance; Prec–Precipitation; MCHO–Monosaccharides; DOC–dissolved organic carbon

* p < 0.05

** p < 0.01.

**Table 5:** Spearman correlation (p) between fluorescence peaks of FDOM, physicochemical parameters and, organic and inorganic nutrients.

		Temp	Sal	Irrad	Prec	MCHO	NO_2_^-^	NO_3_^-^	DOC
α	N1	p = -0.251**	p = -0.509**	p = -.622**	p = 0.615**	p = 0.399**	p = 0.564**	p = 0.557**	p = 0.216*
		(N = 113)	(N = 113)	(N = 113)	(N = 113)	(N = 86)	(N = 100)	(N = 112)	(N = 109)
	I6	p = -0.562**	p = -0.820**	p = -0.720**	p = 0.723**	p = 0.874**	p = 0.481**	p = 0.831**	p = 0.428**
		(N = 114)	(N = 114)	(N = 114)	(N = 114)	(N = 108)	(N = 109)	(N = 113)	(N = 112)
α‘	N1	p = -0.184*	p = -0.500**	p = -0.564**	p = 0.618**	p = 0.402*	p = 0.576**	p = 0.507**	p = 0.232*
		(N = 113)	(N = 113)	(N = 113)	(N = 113)	(N = 86)	(N = 100)	(N = 112)	(N = 109)
	I6	p = -0.544**	p = -0.828**	p = -0.686**	p = 0.738**	p = 0.882**	p = 0.463**	p = 0.805**	p = 0.413**
		(N = 114)	(N = 114)	(N = 114)	(N = 114)	(N = 108)	(N = 109)	(N = 113)	(N = 112)
γ	N1	ϖ = -0.037	p = -0.173	p = -0.248	p = 0.197*	p = 0079	p = 0.060	p = 0.094	p = 0.336**
		(N = 113)	(N = 113)	(N = 113)	(N = 113)	(N = 86)	(N = 97)	(N = 109)	(N = 106)
	I6	p = -0.172	p = -0.554**	p = -0.285**	p = 0.529**	p = 0.564**	p = 0.284**	p = 0.518**	p = 0.422**
		(N = 114)	(N = 114)	(N = 114)	(N = 114)	(N = 103)	(N = 104)	(N = 108)	(N = 107)
β	N1	p = -0.197*	p = -0.435**	p = -0.543**	p = 0.528**	p = 0.428	p = 0.531**	p = 0.469**	p = 0.301**
		(N = 113)	(N = 113)	(N = 113)	(N = 113)	(N = 86)	(N = 100)	(N = 112)	(N = 109)
	I6	p = -0.525**	p = -0.812**	p = -0.698**	p = 0.729**	p = 0.864**	p = 0.460**	p = 0.807**	p = 0.424**
		(N = 114)	(N = 114)	(N = 114)	(N = 114)	(N = 108)	(N = 109)	(N = 113)	(N = 112)
δ	N1	p = 0.264*	p = -0.012	p = 0.124	p = -0.072	p = 0.001	p = -0.010	p = -0.147	p = 0.314**
		(N = 113)	(N = 113)	(N = 113)	(N = 113)	(N = 83)	(N = 96)	(N = 108)	(N = 105)
	I6	p = -0.159	p = -0.542**	p = -0.247	p = 0.568**	p = 0.562**	p = 0.262**	p = 0.497**	p = 0.329*
		(N = 114)	(N = 114)	(N = 114)	(N = 114)	(N = 104)	(N = 106)	(N = 109)	(N = 108)

Temp–Temperature; Sal–Salinity; Irrad–Irradiance; Prec–Precipitation; MCHO–Monosaccharides; DOC–dissolved organic carbon

* p < 0.05

** p < 0.001.

**Table 6:** Spearman correlation (p) between the fluorescence ratios of DOM, physicochemical parameters and, organic and inorganic nutrients.

		Temp	Sal	Irrad	Precip	MCHO	NO_3_^-^	DOC
α‘/α	N1	p = 0.514**	p = 0.223*	p = 0.536**	p = -0.214*	p = 0.042	p = -0.408**	p = 0.020
		(N = 113)	(N = 113)	(N = 113)	(N = 113)	(N = 86)	(N = 112)	(N = 109)
	I6	p = -0.230	p = -0.720**	p = -0.344**	p = 0.665**	p = 0.6865**	p = 0.501**	p = 0.436**
		(N = 114)	(N = 114)	(N = 114)	(N = 114)	(N = 108)	(N = 113)	(N = 112)
γ/α	N1	p_s_ = 0.182	p = 0.243**	p = 0.161	p = -0.208	p = -0.243*	p = -0.286	p = 0.256
		(N = 110)	(N = 110)	(N = 110)	(N = 110)	(N = 85)	(N = 109)	(N = 106)
	I6	p = 0.407**	p = 0.343**	p = 0.516**	p = -0.194*	p = -0.371**	p = -0.327**	p = -0.192*
		(N = 109)	(N = 109)	(N = 109)	(N = 109)	(N = 103)	(N = 108)	(N = 107)
β/α	N1	p = 0.492**	p = 0.472**	p = 0.513**	p = -0.401**	p = -0.196	p = -0.547**	p = 0.035
		(N = 113)	(N = 113)	(N = 113)	(N = 113)	(N = 86)	(N = 112)	(N = 109)
	I6	p = 0.354**	p = 0.095	p = 0.209*	p = 0.171	p = -0.046	p = -0.233*	p = 0.265*
		(N = 114)	(N = 114)	(N = 114)	(N = 114)	(N = 108)	(N = 113)	(N = 112)

Temp–Temperature; Sal–Salinity; Irrad–Irradiance; Precip–Precipitation; MCHO–Monosaccharides; PCHO–Polysaccharides; DOC–dissolved organic carbon

* p < 0.05

** p < 0.001.

### Relationships between optical properties of DOM and environmental factors

**Fig 8:**
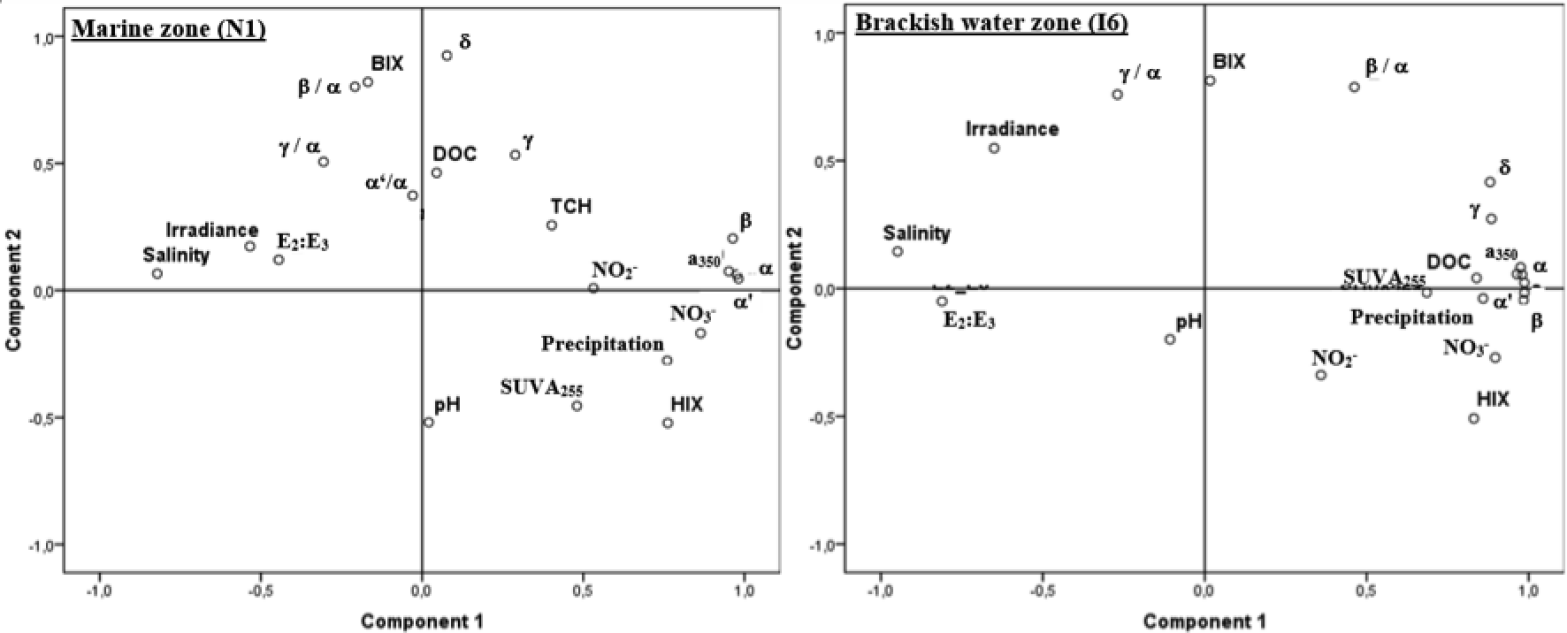
**Principal component analysis of parameters at the marine (A) and brackish water zone (B) of the estuarine system**.

The two components of PCA performed on all variables explained 56.2 and 70.9% of the total variance at the marine (N1) and brackish water (I6) zones of the estuary, respectively (Fig 8). The first component (Factor 1) accounted for 39.7 and 56.7% of the variance at the station N1 and I6, respectively. This component may represent the influence of freshwater inputs, as indicated by its higher loading for salinity. The second component (factor 2) accounted for 16.5 and 14.2% of the total variation at the marine and brackish water zones, respectively. This component characterized the biological productivity at the two estuarine zones, indicated by the γ/α ratio and BIX index. At the brackish water zone (I6), this component was also influenced by solar irradiance.

## Discussion

### Hydrological and biological influences on the dynamics CDOM in the estuary

Salinity [31, 51, 52], freshwater inputs [29] and light [53–55], and their intrinsic seasonal variations are key drivers of biological and chemical processes in estuaries. In the estuarine system Ria de Aveiro, multivariate analysis showed that the variables above mentioned accounted for a higher amount of the CDOM variability at the brackish water zone (approximately 71%) than at the marine zone (approximately 56%). At the brackish water zone, the environmental variables associated with the freshwater inputs, such as higher precipitation, lower salinity and higher nitrate concentration, accounted for nearly 57% of the variability. Consistent and strong correlations between optical properties and these environmental variables are driven by the riverine contribution of CDOM to this estuarine zone. This area is directly impacted by river Boco discharges, in particular, during periods of high flow [29]. A hydrological influence on the CDOM dynamics in estuaries is a common observation [56, 57] and their magnitude and the river flow dependence of CDOM concentration decreases with increased distance from the river mouth [58]. In the present study, a reduced influence of freshwater discharges on CDOM dynamics at the entrance of the estuary was also observed, with freshwater associated variables accounting for less than 40% of the variability. At this estuarine zone, only the optical parameters correlated with land-derived materials, such as the absorption coefficients at 250 nm and 350 nm [20, 21] and, the humic fluorophores α and α’ [16, 47] correlated with the freshwater variables.

The optical properties of CDOM in the Ria de Aveiro were also shaped by biological activity, indicated by BIX and the γ/α ratio. Nevertheless, when compared to freshwater discharges, the influence of biological activity had a lower magnitude, accounting for only nearly 15% of the variability of CDOM at both estuarine zones. The biological index (BIX) is an index of recent autochthonous biological activity that allow to determine the presence of β fluorophore [16]. A strong association of this index to the β fluorophore was observed in the present study, with almost 90% of the variability of this index explained by the ratio β/α. Parlanti et al. [47] carried out algal degradation experiments and showed that the β band could be associated with the degradation of organic materials freshly released by the macro-algae *Ulva lactuca*, which is also present in the Ria de Aveiro, where sometimes shows a high-density biomass [59, 60]. Romera-Castillo et al [61] also observed an increase of this marine-humic peak during the incubation experiments with different axenic phytoplankton cultures and extrapolated that about 20% of the marine humic–like substances produced in the coastal upwelling system of the Ría de Vigo could originate from phytoplankton. Previous studies, carried out in estuaries, also showed that the β fluorophore is not only present in marine waters, but also in freshwaters, and is associated with small molecules [62]. Although the BIX index and the ratio β/α presented a small variation at the brackish water zone, a slight increase during the wet period (group 4) was observed. Previous studies [29] showed that phytoplankton biomass at this particular estuarine zone during the periods of high flow is supplied by the river Boco, which might contribute to the slight increase of these biological activity indicators. The highest values of BIX index and the ratio β/α were observed however, during the dry period at both estuarine zones, and were directly correlated to temperature, salinity and irradiation. These are key factors that stimulate the primary productivity in estuaries [55] and in the case of Ria de Aveiro, contribute to a huge stimulation of the phytoplankton activity at marine zone, where the differences between the hot and cold seasons could reach 112 times [51]. Another indicator of recent autochthonous productivity, the γ fluorophore, is associated with labile compounds of protein or bacterial origin [63]. In Ria de Aveiro, the total amount of this fluorophore was related to the freshwater inputs. However, its relative abundance to the α fluorophore (the ratio γ/α) was stimulated by temperature, salinity and solar irradiance, which increase during the dry period. The dynamics of DOM in Ria de Aveiro are therefore highly impacted by the freshwater inputs in the system and, during the hot and dry seasons, influenced as well by the autochthone productivity, stimulated by an increase of solar irradiance and temperature. These observations support previous findings about the importance of freshwater discharges [29] and the role of allochthonous organic matter [64] in the productivity of the estuarine system Ria de Aveiro.

### Seasonal variations of the CDOM in the estuary

The spatial variations of optical characteristics in estuaries are well described in the literature [16, 62]; however few studies [65, 66] have addressed the seasonality in these complex ecosystems. In order to further interpret the results in terms of the seasonal variability of CDOM in the estuarine system Ria de Aveiro, meteorological and water column properties were measured during the extensive two-year survey and it was observed that some of those properties (precipitation, irradiance and salinity) could be used to group the numerous sampling events into four organised categories (groups), in order to better explain the variability of nutrients and DOM along time. Group 4 (with a small number of samples) is more affected by extreme rainy events and the group 1 (with a high number of samples) by the dry weather. Extreme rainy events (sampling events from group 4) occurred during winter. Dry weather, high irradiance and high salinity were more frequent during summer and autumn seasons. Thus, samples from groups 1 and 2 are mainly from these seasons. However, some sampling events from winter or spring corresponded to dry weather and sunny days, reason why a few of those events were allocated to groups 1 or 2.

In Ria de Aveiro, the concentration of DOC, carbohydrates and inorganic nutrients showed a tendency to increase with precipitation. This observation supports previous proposed hypotheses on the relevance of runoff events and freshwater inputs for the dynamics of DOM in the estuary. Former studies showed that the runoff from flooded margins contributes significantly with DOC to the estuary [51]. During the wet season, high freshwater inputs also contribute with DOC and inorganic nutrients to the estuary [29, 64, 67]. Chromophoric and fluorescent fractions of DOM followed a similar tendency, showing particularly high concentrations during storm events and periods of elevated freshwater inflow in the system, as represented by group 4. During this period, the concentration of CDOM (a_250_ and a_350_), its aromaticity (SUVA_254_) and humic content (α and α´) increased significantly, in particular, at the marine zone. Simulations showed that under a scenario of maximum freshwater inflow in the estuary, such as those of group 4, the residence time of water masses in the Ílhavo channel is less than four days [29]. Dixon et al. [58] observed that flow rate and residence time are among the most important factors influencing diagenetic alterations of DOM within an estuary. A rapid transport through the system will hamper a substantial processing and alteration of CDOM by mechanisms such *in situ* production [47, 68, 69], photobleaching [23, 70], particle adsorption and/or flocculation [71]. Therefore, storm events and high freshwater inflow to the estuarine system produce particular hydrological conditions, which promote the conservative transport of CDOM downstream. Under this hydrological environment, the optical properties of CDOM at the mouth, even diluted, will be more similar to those at the upper estuary.

In contrast, during the dry periods, such as those where group 1 events clustered, the residence time of water masses in the Ílhavo channel is very long. Previous simulations showed that particles released from the brackish water zone station remained in upper zone of the Ílhavo channel after 360–432 h [29]. A long residence time of water masses, allied to the increase of temperature and solar irradiance, will stimulate the microbial activity, promoting the bioprocessing of CDOM within the estuary. Additionally, a long residence time will also increase time exposure of CDOM to solar irradiance, and its consequent photobleaching. In both sampling sites, CDOM within this group showed the lowest values of aromaticity (SUVA_254_), and humification (HIX). This reduction of the aromatic moieties of DOM was associated with an increase of the biological index (BIX) and the ratio β/α and a decrease of the average molecular weight of DOM (increase of the ratio E_2_:E_3_). Helms et al. [23] observed that photobleaching of CDOM results in clear fluorescent trends, with increased β/α ratio and BIX, while HIX decreased significantly. Moreover, the photochemical processes can significantly reduce the average molecular size of DOM, destroying the high-molecular-weight components and forming low-molecular-weight compounds [17, 72], explaining the increase of ratio E_2_:E_3_. Therefore, a long residence time of water masses associated with a seasonal increase in solar irradiance will produce particular conditions to the occurrence of photochemical processes, leading to mineralization and transformation of terrestrial DOM within the estuary. A CDOM photo-transformation during seaward transport is supported by an average value of the ratio β/α > 1, in the group 1 in the marine zone, which indicates an extensively bleached terrestrial CDOM [23, 24]. Nevertheless, a high β/α ratio could be an indication of algal and/or microbial productivity as well [24, 47, 73]. During the hot and dry season, the overall biomass productivity increases in the estuary, reaching its maximum at the marine zone and at the high tide [51]. Both bacterial and phytoplankton activities can produce chromophoric [74, 75] and fluorescent DOM [69, 76], influencing the dynamics of its optical properties. Bacterial incubations of DOM from different sources can result in an increase of the values of the SUVA and the HIX index [75]. In this work, only a slight increase of the HIX at the marine zone was observed during the events that clustered in group 1, which could be related to the microbial processing of algal-derived DOM. Guillemette and del Giorgio [76] also reported the production of humic-like fractions during incubations of bacteria with DOM, with rates varying as a function of bacterial growth efficiency and concentration of inorganic nutrients. Net production and consumption of DOM by bacteria growing on phytoplankton exudates from different species was also observed by Romera-Castillo et al. [69], with bacteria producing humic-like alpha fluorophore. Thus, periods of high microbiological activity can cause significant alterations in the CDOM pool, producing modifications that resemble those of photochemical reactions and making DOM source inferences during the dry period a very hard task. Moreover, the deepness of biological imprints on DOM pool might be dependent on the seasonal and inter-annual quantities of nutrients inputs in the system and on solar irradiance.

## Conclusions

The dynamics of CDOM in the estuarine system Ria de Aveiro is mainly influenced by the hydrological conditions, and their influence decreases as the oceanic influence increases seaward. Land-derived compounds are the predominant source of CDOM in the estuary during almost all the year. However, biological activity during the dry and hot season also produces traceable transformations of DOM in the estuarine system, with strongest impressions in the oceanic influenced area. The results of this study showed clearly that BIX index and α and α' fluorescence peaks can be used as sensitive and specific tracers of DOM origin in biogeochemical studies. The factor loading plot for PCA Component 1 and Component 2 shows that the fluorescence indices are the most reliable and specific for tracing terrestrial and marine DOM, respectively. BIX appears to be a robust indicator of marine type DOM (it is almost entirely carried by Component 2 which is associated with the marine influence) and α and α' fluorescence peaks seem equally good tracers of terrestrial DOM (it is almost entirely carried by Component 1 which is associated with freshwater inputs). These findings, made possible by the experimental design of this study, covering an extensive sampling period, may be used in other studies to trace the origin of DOM.

Based on the spatial and seasonal variability, it is expectable that CDOM photo-reactivity will be also different, with a greater amount of photo-induced reactions occurring in the up-estuary and during the wet season. Expected climate changes related to seasonal and inter-annual variations of the precipitation amounts might impact the dynamics of CDOM significantly, influencing its photochemistry and the microbiological activities in estuarine systems.

## Supporting Information

S1 Table Concentration of monosaccharides (MCHO) and polysaccharides (PCHO), dissolved inorganic nitrogen species (ammonium (NH4^+^), nitrite (NO2^-^) and nitrate (NO3^-^) and dissolved organic carbon (DOC) within the different groups at the marine (N1) and brackish water (I6) zones in the estuarine system Ria de Aveiro. (DOCX)

S2 Table Variation within the groups of the CDOM absorption coefficients at 250 (a_250_) and 350 nm (a_350_), of the ratio E_2_:E_3_ and of the specific ultra-violet absorbance at 254 nm (SUVA_254_), at the marine (N1) and brackish water (I6) zones of the estuarine system Ria de Aveiro. (DOCX)

S3 Table Variation within the groups of the major FDOM components at the marine (N1) and brackish water (I6) zones of the estuarine system Ria de Aveiro. (DOCX)

S4 Table Variation within the groups of the FDOM ratios and indexes at the marine (N1) and brackish water (I6) zones of the estuarine system Ria de Aveiro. (DOCX)
