# Supplementary material for: Insights on the Optical Properties of Estuarine DOM – Hydrological and Biological Influences
Source: PLoS One. 2016 May 19;11(5):e0154519. doi: 10.1371/journal.pone.0154519 (PMC4873235; doi:10.1371/journal.pone.0154519)
Supplement: S1 Table — (DOCX) [file pone.0154519.s001.docx]

S1 Table. Concentration of monosaccharides (MCHO) and polysaccharides (PCHO), dissolved inorganic nitrogen species (ammonium (NH_4_^+^), nitrite (NO_2_^-^) and nitrate (NO_3_^-^) and dissolved organic carbon (DOC) within the different groups at the marine (N1) and brackish water (I6) zones in the estuarine system Ria de Aveiro.

| Groups | 1 | 2 | 3 | 4 |
| --- | --- | --- | --- | --- |
| Marine zone (N1) | | | | |
| MCHO | 15.8 ± 6.7 | 17.8 ± 6.5 | 22.1 ± 6.4 | 42.6 ± 3.8 |
| (µM.C) | (< LOD – 32.1) | (< LOD – 30.3) | (12.8 – 32.2) | (38.4 – 46.3) |
|  | N=45 (3) | N=30 (4) | N=18 (3) | N=4 (0) |
| PCHO | 11.7 ± 7.2 | 14.7 ± 8.2 | 8.1 ± 5.7 | 12.0 ± 1.1 |
| (µM.C) | (1.8 – 39.8) | (4.0 – 33.5) | (0.44 – 18.3) | (10.6 – 13.2) |
|  | N=37 (0) | N=25 (0) | N=13 (0) | N=4(0) |
| NH_4_^+^ | 3.2 ± 1.9 | 2.28 ± 0.94 | 3.7 ± 1.5 |  |
| (µM) | (1.1 – 8.1) | (0.92 – 3.95) | (1.9 – 5.4) |  |
|  | N=48 (0) | N=29 (0) | N=12 (0) | N.D. |
| NO_2_^-^ | 0.43 ± 0.24 | 0.35 ± 0.24 | 0.73 ± 0.30 | 0.940 ± 0.050 |
| (µM) | (< LOD – 0.94) | (< LOD – 0.95) | (0.37 – 1.13) | (0.860 – 0.980) |
|  | N=54 (2) | N=33 (9) | N=21 (0) | N=4 (0) |
| NO_3_^-^ | 17.8 ± 7.9 | 10.9 ± 5.7 | 26.2 ± 7.3 | 69.7 ± 3.9 |
| (µM) | (6.4 – 35.7) | (4.2 – 23.8) | (14.4 – 41.9) | (65.7 – 74.7) |
|  | N=55 (0) | N=33 (0) | N=22 (0) | N=4 (0) |
| DOC | 3.9 ± 1.9 | 2.4 ± 1.6 | 3.3 ± 2.1 | 3.51 ± 0.41 |
| (mg L^-1^) | (1 – 8.9) | (0.8 – 7.2) | (1.3 – 7.5) | (3.15 – 3.95) |
|  | N=51 (0) | N=34 (0) | N=22 (0) | N=4 (0) |
| Brackish water zone (I6) | | | | |
| MCHO | 50 ± 11 | 72 ± 21 | 89 ± 27 | 215 ± 76 |
| (µM.C) | (23 – 71) | (46 – 119) | (55 – 123) | (124 – 354) |
|  | N=55 (0) | N=28 (0) | N=13 (0) | N=13 (0) |
| PCHO | 35 ± 115 | 26 ± 11 | 28 ± 14 | 54 ± 15 |
| (µM.C) | (8 – 64) | (2 – 52) | (4 – 45) | (34 – 86) |
|  | N=55 (0) | N=26 (0) | N=13 (0) | N=12 (0) |
| NH_4_^+^ | 6.7 ± 1.9 | 7.6 ± 2.3 | 11.47 ± 0.64 | 8.6 ± 3.3 |
| (µM) | (3.8 – 10.4) | (5.2 – 12.5) | (10.70 – 12.28) | (5.0 – 12.0) |
|  | N=52 (0) | N=28 (0) | N=5 (0) | N=9 (0) |
| NO_2_^-^ | 1.13 ± 0.34 | 1.41 ± 0.46 | 2.7 ± 1.1 | 1.87 ± 0.72 |
| (µM) | (< LOD – 1.84) | (0.78 – 2.31) | (1.5 – 3.8) | (1.12 – 2.80) |
|  | N=56 (4) | N=28 (0) | N=13 (0) | N=13 (0) |
| NO_3_^-^ | 10.0 ± 2.8 | 46 ± 12 | 39 ± 17 | 91 ± 18 |
| (µM) | (5.2 – 17.0) | (30 – 63) | (23 – 63) | (74 – 119) |
|  | N=60 (0) | N=28 (0) | N=13 (0) | N=13 (0) |
| DOC | 5.9 ± 1.8 | 6.5 ± 2.1 | 6.9 ± 2.4 | 13.3 ± 3.3 |
| (mg L^-1^) | (3.8 – 12.1) | (4.3 – 12.0) | (3.8 – 10.2) | (8.9 – 16.6) |
|  | N=60 (0) | N=26 (0) | N=13 (0) | N=13 (0) |

N.D. not determined;

Average ± standard deviation; (range of variation); number of samples analysed (number of samples <LOD)
