# Supplementary material for: Insights on the Optical Properties of Estuarine DOM – Hydrological and Biological Influences
Source: PLoS One. 2016 May 19;11(5):e0154519. doi: 10.1371/journal.pone.0154519 (PMC4873235; doi:10.1371/journal.pone.0154519)
Supplement: S2 Table — (DOCX) [file pone.0154519.s002.docx]

S2 Table. Variation within the groups of the CDOM absorption coefficients at 250 (a_250_) and 350 nm (a_350_), of the ratio E_2_:E_3_ and of the specific ultra-violet absorbance at 254 nm (SUVA_254_), at the marine (N1) and brackish water (I6) zones of the estuarine system Ria de Aveiro.

| Group | 1 | 2 | 3 | 4 |
| --- | --- | --- | --- | --- |
| Marine zone (N1) | | | | |
| a_250_ | 4.08 ± 0.45 | 4.06 ± 0.59 | 5.2 ± 1.1 | 16.32 ± 0.21 |
| (m^-1^) | (3.29 – 4.94) | (3.21 – 4.96) | (4.0 – 7.7) | (16.12 – 16.58) |
|  | N=54 | N=34 | N=22 | N=4 |
| a_350_ | 0.74 ± 0.11 | 0.74 ± 0.17 | 1.06 ± 0.26 | 3.940 ± 0.060 |
| (m^-1^) | (0.53 – 1.03) | (0.46– 1.01) | (0.80 – 1.66) | (3.870 – 4.010) |
|  | N=54 | N=34 | N=22 | N=4 |
| E_2_:E_3_ | 7.07 ± 0.48 | 6.49 ± 0.39 | 6.61 ± 0.58 | 5.410 ± 0.050 |
|  | (5.93 – 8.26) | (5.81 – 7.52) | (5.77 – 7.83) | (5.370– 5.480) |
|  | N=54 | N=34 | N=22 | N=4 |
| SUVA_254_ | 0.52 ± 0.25 | 0.98 ± 0.37 | 0.88 ± 0.41 | 1.76 ± 0.41 |
| (L mg^-1^ C^-1^ m^-1^) | (0.19 – 1.20) | (0.25 – 1.71) | (0.26 – 1.44) | (1.20 – 2.13) |
|  | N=51 | N=34 | N=22 | N=4 (0) |
| Brackish water zone (I6) | | | | |
| a_250_ | 23.3 ± 3.1 | 28.6 ± 4.6 | 36.7 ± 8.3 | 102 ± 28 |
| (m^-1^) | (18.5 – 29.8) | (22.1 – 37.3) | (26.5 – 43.6) | (68 – 132) |
|  | N=61 | N=28 | N=13 | N=13 |
| a_350_ | 4.90 ± 0.64 | 6.0 ± 1.2 | 8.3 ± 1.8 | 25.1 ± 7.6 |
| (m^-1^) | (3.79 – 6.25) | (4.4 – 8.4) | (6.2 – 10.0) | (16.0– 33.2) |
|  | N=61 | N=28 | N=13 | N=13 |
| E_2_:E_3_ | 6.11 ± 0.16 | 6.29 ± 0.24 | 5.71 ± 0.16 | 5.30 ± 0.10 |
|  | (5.47 – 6.37) | (5.78 – 6.71) | (5.46 –5.96) | (5.17 – 5.42) |
|  | N=61 | N=28 | N=13 | N=13 |
| SUVA_254_ | 1.78 ± 0.54 | 1.91 ± 0.48 | 2.32 ± 0.39 | 3.18 ± 0.24 |
| (L mg^-1^ C^-1^ m^-1^) | (0.82 – 2.71) | (1.20 – 2.66) | (1.75 – 2.95) | (2.90 – 3.59) |
|  | N=60 | N=26 | N=13 | N=13 |
