# Supplementary material for: Insights on the Optical Properties of Estuarine DOM – Hydrological and Biological Influences
Source: PLoS One. 2016 May 19;11(5):e0154519. doi: 10.1371/journal.pone.0154519 (PMC4873235; doi:10.1371/journal.pone.0154519)
Supplement: S3 Table — (DOCX) [file pone.0154519.s003.docx]

**S3 Table.** Variation within the groups of the major FDOM components at the marine (N1) and brackish water (I6) zones of the estuarine system Ria de Aveiro.

| Group | | | 1 | 2 | | | 3 | 4 | | |
| --- | --- | --- | --- | --- | --- | --- | --- | --- | --- | --- |
| Marine zone (N1) | | | | | | | | | | |
| α | | | 26.0 ± 3.5 | 22.1 ± 2.8 | | | 41 ± 13 | 123.1 ± 1.5 | | |
| (QSU) | | | (21.0 – 35.0) | (17.0 – 26.0) | | | (27 – 70) | (122.0 – 125.0) | | |
|  | N=54 | | | N=33 | N=22 | | | N=4 |  |  |
| α’ | | | 61.9 ± 8.4 | 53.3 ± 6.7 | | | 95 ± 32 | 302.4 ± 4.8 | | |
| (QSU) | | | (50.0 – 81.0) | (41.0 – 61.0) | | | (61 – 162) | (298.0 – 308.0) | | |
|  | N=54 | | | N=33 | N=22 | | | N=4 |  |  |
| β | | | 26.7 ± 5.4 | 21.4 ± 3.0 | | | 38 ± 11 | 108.4 ± 1.8 | | |
| (QSU) | | | (20.0 – 41.0) | (17.0 – 26.0) | | | (26 – 61) | (107.0 – 111.0) | | |
|  | N=54 | | | N=33 | N=22 | | | N=4 |  |  |
| γ | | | 24 ± 15 | 17 ± 10 | | | 24 ± 15 | 44.7 ± 3.8 | | |
| (QSU) | | | (7 – 59) | (7 – 38) | | | (8 – 55) | (42.0 – 50.0) | | |
|  | N=54 (1)* | | | N=33 (1)* | N=22 (1)* | | | N=4 |  |  |
| δ | | | 43 ± 33 | 30.6 ± 7.5 | | | 27.1 ± 8.7 | 53.0 ± 3.2 | | |
| (QSU) | | | (16 – 159) | (23.0 – 57.0) | | | (20.0 – 51.0) | (51.0 – 57.0) | | |
|  | N=54 (2)* | | | N=33 (1)* | N=22 | | | N=4 |  |  |
| Brackish water zone (I6) | | | | | | | | | | |
| α | | | 135 ± 15 | 183 ± 24 | | | 229 ± 59 | 402 ± 90 | | |
| (QSU) | | | (113 – 180) | (139 – 210) | | | (157 – 282) | (288 – 482) | | |
|  | N=60 | | | N=28 | N=13 | | | N=13 |  |  |
| α’ | | | 387 ± 50 | 539 ± 87 | | | 710 ± 219 | 1668 ± 477 | | |
| (QSU) | | | (311 – 541) | (382 – 664) | | | (444 – 931) | (1061 – 2120) | | |
|  | N=60 | | | N=28 | N=13 | | | N=13 |  |  |
| β | | | 129 ± 14 | 171 ± 22 | | | 216 ± 56 | 391 ± 92 | | |
| (QSU) | | | (108 – 168) | (129 – 195) | | | (147 – 268) | (274 – 466) | | |
|  | N=60 | | | N=28 | N=13 | | | N=13 |  |  |
| γ | | | 53 ± 15 | 57 ± 14 | | | 78 ± 24 | 126 ± 35 | | |
| (QSU) | | | (31 – 88) | (39 – 84) | | | (45 – 102) | (81 – 182) | | |
|  | N=60 (2)* | | | N=28 (2)* | N=13 (1)* | | | N=13 |  |  |
| δ | | | 101 ± 33 | 103 ± 14 | | | 133 ± 27 | 225 ± 45 | | |
| (QSU) | | | (65 – 248) | (77 – 133) | | | (91 – 154) | (166 – 304) | | |
|  | N=60 (4)* | | | N=28 | N=13 | | | N=13 |  |  |

* (number of data rejected due to abnormalities in this region of the spectra)
