# Supplementary material for: Insights on the Optical Properties of Estuarine DOM – Hydrological and Biological Influences
Source: PLoS One. 2016 May 19;11(5):e0154519. doi: 10.1371/journal.pone.0154519 (PMC4873235; doi:10.1371/journal.pone.0154519)
Supplement: S4 Table — (DOCX) [file pone.0154519.s004.docx]

S4 Table. Variation within the groups of the FDOM ratios and indexes at the marine (N1) and brackish water (I6) zones of the estuarine system Ria de Aveiro.

| Group | 1 | 2 | 3 | 4 |
| --- | --- | --- | --- | --- |
| Marine zone (N1) | | | | |
| α’/α | 2.380 ± 0.060  (2.250-2.550)  N=54 | 2.410 ± 0.040  (2.360-2.520)  N=33 | 2.300 ± 0.040  (2.240-2.400)  N=22 | 2.460 ± 0.010  (2.450-2.470)  N=4 |
| γα | 0.91 ± 0.55  (0.33-2.28)  N=53 | 0.81 ± 0.51  (0.29-1.82)  N=32 | 0.59 ± 0.34  (0.27-1.39)  N=21 | 0.360 ± 0.030  (0.340-0.400)  N=4 |
| β/α | 1.03 ± 0.15  (0.92-1.51)  N=54 | 0.970 ± 0.030  (0.920-1.070)  N=33 | 0.940 ± 0.039  (0.870-1.000)  N=22 | 0.880 ± 0.010  (0.880-0.890)  N=4 |
| HIX | 2.9 ± 1.1  (0.9-4.8)  N=52 | 2.69 ± 0.37  (1.84-3.29)  N=33 | 5.3 ± 1.5  (3.4-8.2)  N=21 | 8.12 ± 0.43  (7.63-8.49)  N=4 |
| BIX | 0.95 ± 0.36  (0.75-2.24)  N=54 | 0.850 ± 0.090  (0.770-1.180)  N=33 | 0.740 ± 0.050  (0.660-0.850)  N=22 | 0.680 ± 0.010  (0.670-0.690)  N=4 |
| Brackish water zone (I6) | | | | |
| α’/α | 2.850 ± 0.070  (2.730-3.010)  N=60 | 2.93 ± 0.13  (2.75-3.16)  N=28 | 3.06 ± 0.20  (2.81-3.30)  N=13 | 4.09 ± 0.32  (3.69-4.48)  N=13 |
| γ/α | 0.390 ± 0.090  (0.250-0.650)  N=58 | 0.310 ± 0.070  (0.230-0.460)  N=26 | 0.330 ± 0.030  (0.280-0.370)  N=12 | 0.310 ± 0.030  (0.280-0.390)  N=13 |
| β/α | 0.960 ± 0.020  (0.930-1.080)  N=60 | 0.93 ± 0.00  (0.93-0.94)  N=28 | 0.940 ± 0.010  (0.930-0.960)  N=13 | 0.970 ± 0.020  (0.950-0.990)  N=13 |
| HIX | 6.0 ± 1.4  (2.0-8.2)  N=59 | 8.5 ± 1.2  (5.9-10.2)  N=27 | 8.2 ± 1.1  (5.4-9.1)  N=13 | 11.9 ± 1.2  (10.2-13.4)  N=13 |
| BIX | 0.790 ± 0.080  (0.730-1.230)  N=60 | 0.730 ± 0.010  (0.730-0.740)  N=28 | 0.750 ± 0.010  (0.740-0.770)  N=13 | 0.770 ± 0.010  (0.760-0.780)  N=13 |
